# Mapping macaque to human cortex with natural scene responses

**DOI:** 10.1073/pnas.2512619122

**Published:** 2025-09-30

**Authors:** Kasper Vinken, Saloni Sharma, Margaret S. Livingstone

**Affiliations:** ^a^Department of Neurobiology, Harvard Medical School, Boston, MA 02115

**Keywords:** functional correspondence, face processing, natural scenes, monkey, human

## Abstract

Neuroscience has long relied on macaque studies to infer human brain function, yet identifying functionally corresponding brain regions across species and measurement modalities remains a fundamental challenge. This is especially true for the higher-order cortex, where functional interpretations are constrained by narrow hypotheses and anatomical landmarks are often nonhomologous. We present a data-driven approach for mapping functional correspondence across species using rich, naturalistic stimuli. By directly comparing macaque electrophysiology with human fMRI responses to 700 natural scenes, we identify fine-grained alignment based on response pattern similarity, without relying on predefined tuning concepts or hand-picked stimuli. As a test case, we examine the ventral face patch system, a well-studied but contested domain in cross-species alignment. Our approach resolves a long-standing ambiguity by supporting a correspondence between macaque ML and human FFA and between AL and more anterior temporal cortex in humans. This result is consistent with full-brain anatomical warping but inconsistent with prior studies limited by narrow functional hypotheses. These findings show that natural image-evoked response patterns provide a robust foundation for cross-species functional alignment, supporting scalable comparisons as large-scale primate recordings become more widespread.

## Significance

Understanding how human and nonhuman primate brains are related is central to neuroscience, but matching brain areas across species remains challenging. Traditional methods rely on simplified interpretations of what each area does, limiting their ability to reveal similarities. For example, regions that respond well to faces are typically studied using only face images. We introduce a data-driven approach that compares how human and monkey brains respond to hundreds of natural images, without relying on predefined categories or functional labels. Using this method, we show that key macaque face-processing areas align with more anterior regions in the human brain than often assumed. Our findings show that shared brain organization can be uncovered through naturalistic stimulation, without assuming what each region represents.

Neuroscience depends on cross-species and cross-measurement comparisons to understand the principles of human brain function. Research in humans is mostly based on noninvasive techniques such as functional MRI (fMRI), which measures broad, indirect signals throughout the entire brain. This technique has been widely used to localize function to the cortex, for instance, to identify visual regions with response biases for edges, shapes, faces, bodies, or other categories (1–6). Disentangling the neural computations in these regions requires more focal recording techniques that are largely restricted to animal models. Therefore, much of our understanding of human visual processing has been inferred indirectly from electrophysiological measures in the macaque visual system. A central challenge for systems neuroscience is to connect those findings by aligning responses across measurement modalities and identifying functionally corresponding brain regions across species. Establishing such correspondence is not only critical for interpreting human brain function based on findings from animal models but also provides insights into the evolutionary organization of the cortex (7).

Existing approaches often rely on narrowly defined functional hypotheses or a small number of diagnostic stimuli to infer correspondence (8–13). But such features may fail to capture the full complexity of cortical representations. Even in the early visual cortex, where simple stimuli have yielded a clear and interpretable picture of neural responses, this understanding often breaks down under naturalistic stimulus conditions (14, 15). These limitations are likely even more pronounced in higher-order areas, where representations are shaped by more complex features and conceptual distinctions between regions remain poorly understood (16). As large-scale, brain-wide recordings in nonhuman primates become more widely available (17), and as neuroscience expands into more abstract cortical territories, these limitations become more acute, highlighting the need for scalable, hypothesis-agnostic methods that can leverage such data. Here, we propose one such framework: a flexible, data-driven approach for identifying functionally corresponding brain regions across species using response selectivity to a large number of naturalistic stimuli. By leveraging the rich, graded response patterns evoked by diverse natural scenes, this method identifies functional alignment without relying on predefined tuning axes, stimulus categories, or conceptual heuristics.

To evaluate this approach, we focus on the ventral face patch system, a well-characterized yet still unresolved test case for cross-species alignment. This system comprises a series of patches that respond more to faces than to other objects [“face selectivity” (3, 18)]. In humans, the ventral face patches form a sequence that extends posteriorly from the occipital face area (OFA) through the fusiform face area’s posterior (FFA-1) and anterior parts (FFA-2) and culminates anteriorly at the anterior temporal lobe face patch (ATL). In the macaque inferotemporal cortex (IT), a comparable sequence unfolds from the posterior lateral face patch (PL) through the middle lateral (ML) and anterior lateral face patches (AL), leading to the anterior medial face patch (AM) (8, 9). These patches contain a high proportion of face-selective neurons called “face cells”, which have been studied extensively in the macaque visual cortex (18–36). While both species exhibit a broadly conserved posterior-to-anterior axis, the precise correspondence of specific patches remains unresolved (9). The macaque central IT face patch ML and human FFA are often considered homologous (8, 37–39). This notion is supported by predictions based on full-brain anatomical warping, which suggests an ML to FFA and an AL to ATL correspondence (39, 40). However, evidence from the surrounding cortical topography suggests a possible PL to OFA, ML to FFA-1, and AL to FFA-2 correspondence (41) or even a PL/ML to OFA and AL to FFA correspondence (10, 42).

To further complicate the picture, anatomical evidence may be insufficient on its own to establish the correspondence of face areas, as regions can reorganize, duplicate, segregate, or enlarge through cortical expansion during evolution (43–45). Functional evidence is therefore essential to determine whether two regions in macaques and humans serve similar roles in visual processing. Traditionally, functional correspondence is determined based on a conceptual interpretation of the role that a region may serve in brain processing. For macaque face regions, such a conceptual distinction is how neurons respond to heads presented at different orientations. Whereas ML neurons show viewpoint-specific responses, more anterior AL neurons show mirror-symmetric viewpoint invariance (22), a tuning property that can be retrieved from fMRI activation patterns (11). In humans, one study found evidence for this mirror-symmetric coding in FFA, but not in OFA (46), suggesting an ML to OFA and AL to FFA functional correspondence. An alternative interpretation is that ML may correspond to the more posterior part FFA-1 and AL to the more anterior part FFA-2 (9). Others have suggested that evidence for mirror-symmetric tuning could merely be explained by low-level confounds (47, 48). Causal studies may offer additional evidence of functional distinctions (24, 49). However, they too are typically restricted to low-dimensional, conceptual characterizations, such as involvement in face detection or identity recognition, and remain sparse in humans. The lack of consistency among studies calls into question the validity of relying on a single, hand-picked tuning property or conceptual distinctions to arbitrate between scenarios of functional correspondence across species. Indeed, recent findings have shown that, even for face cells, the neural tuning should not be reduced to a few human-interpretable features or concepts (35, 36), but instead reflects complex, distributed representations best captured through more varied, naturalistic stimulation.

Here, we test whether we can establish the functional correspondence of human and macaque face areas without relying on specific assertions of functional specialization. Rather than focusing on only face images or predefined functional properties, we compared neural responses from macaque and human cortex to a shared, diverse set of natural scenes. Specifically, we leveraged an existing large-scale dataset of human fMRI responses to complex natural scenes (50) and recorded neural responses in macaque central and anterior face regions using the same stimuli. This allowed us to evaluate whether response selectivity across a large number of natural images can resolve competing hypotheses about the alignment of face-selective regions across species. We found that a direct comparison of these responses supported a mapping in which macaque ML corresponds to human FFA and AL to ATL, consistent with predictions from full-brain anatomical warping alone (39). Thus, our approach resolved prior inconsistencies and demonstrated that broad, natural image-evoked response profiles can establish functional alignment across species, without assuming conceptual distinctions between regions.

## Results

**Fig. 1.**
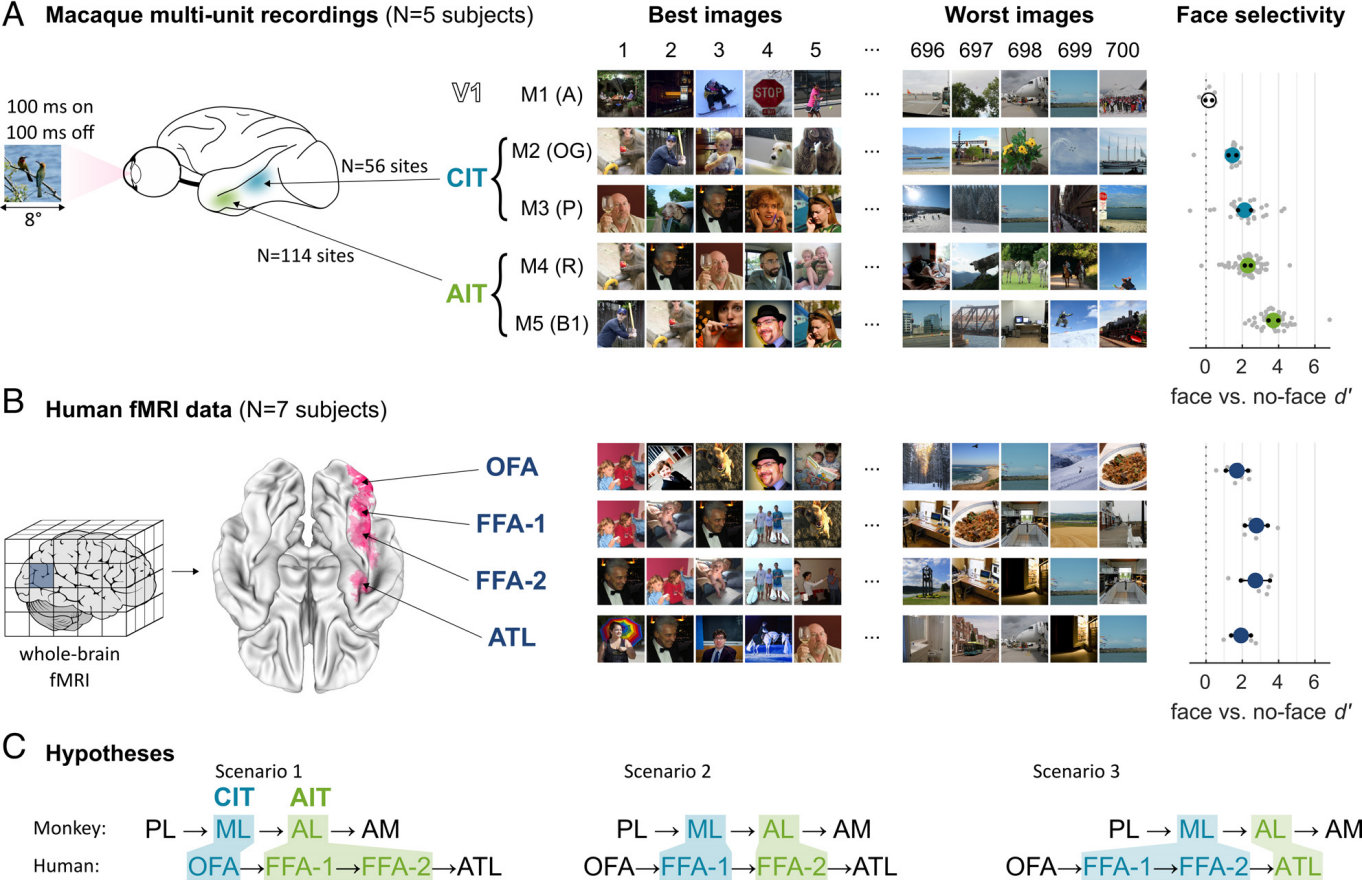
Data and hypotheses. (*A*) Central and anterior IT array locations show face-selective responses. *Middle*: images ranked according to the array response averaged across units. *Right*: average face selectivity. Gray markers represent individual units. Black markers indicate 95% CI. (*B*) Human face-selective ROIs from the functional localizer experiment of (50). *Middle*: images ranked according to the ROI response averaged across voxels and subjects. *Right*: average ROI face selectivity. Gray markers represent individual subjects. Black markers indicate 95% CI. (*C*) Three scenarios of posterior–anterior functional alignment of face-selective cascade in the macaque and human ventral streams.

We presented stimuli from the Natural Scenes Dataset (NSD) (50, 51) to five macaque monkeys (initials A, OG, P, R, and B1; hereafter referred to as M1–M5), one with an array in the primary visual cortex (V1; M1: N = 11 reliably responsive multiunit sites, see *Methods*), two with arrays in CIT at the location of the middle lateral face patch (ML; M2: N = 23 units; M3: N = 33 units), and two with arrays in AIT targeting the anterior lateral face patch (AL; M4: N = 74 units; M5: N = 40 units). The stimulus set consisted of a rich set of 700 photographs of a variety of things in a scene context (including animals, humans, sports, food…) captured at varying distances. Most of these stimuli did not prominently feature faces. On average, each of the IT arrays (but not the V1 array) responded most strongly to images with prominent faces (Fig. 1*A*). To evaluate face selectivity of individual units, we computed a face versus no-face d’ metric comparing the response to images with prominent faces to those without faces or animals (*Methods*). The units recorded from IT arrays had a high level of face selectivity [mean face versus no-face d’; M2, CIT: 1.46, 95% CI(1.24,1.67); M3, CIT: 2.12, 95% CI(1.76,2.48); M4, AIT: 2.30, 95% CI(2.14,2.47); M5, AIT: 3.71, 95% CI(3.44,3.98)], unlike the V1 array [M1: 0.15, 95% CI(−0.03,0.34)] (52).

Similarly, for the human fMRI data, all four localized face area regions of interest (ROIs) responded most strongly to images with prominent faces (Fig. 1*B*). Note that we excluded the midtemporal lobe face ROI, since it was localized in only two subjects. We computed a face versus no-face d’ per ROI of each individual subject, confirming that these regions have strongly face-selective responses [mean face versus no-face d’; OFA: 1.70, 95% CI(1.09,2.31); FFA-1: 2.78, 95% CI(2.15,3.40); FFA-2: 2.72, 95% CI(1.92,3.52); ATL: 1.93, 95% CI(1.38,2.47)]. Thus, the CIT and AIT arrays were highly face-selective, like human face areas, confirming that we successfully targeted the middle and anterior face-selective regions in the IT cortex.

To determine whether response patterns across natural scenes can resolve the correspondence between macaque and human face-selective regions, we evaluated three hypothesized alignments (Fig. 1*C*): 1) ML to OFA and AL to FFA; 2) ML to FFA-1 and AL to FFA-2; 3) ML to FFA and AL to ATL. That is, does macaque ML correspond best to human OFA (scenario 1) or to (posterior) human FFA (scenario 2 or 3), and does macaque AL correspond best to human (anterior) FFA (scenario 1 or 2) or to human ATL (scenario 3)? We used array-to-fMRI response pattern similarity to arbitrate between these scenarios.

### Macaque IT Arrays Correlate With Human fMRI Face Areas and Beyond.

Having established the face selectivity of the monkey arrays, where in the human brain do we find similar tuning? Rather than characterizing and interpreting the tuning of neural or fMRI responses as a function of specific visual features or categories, we take an agnostic approach where we compare the full selectivity profile across all images. This approach is motivated by recent work from our lab, where we showed that the tuning of face cells is more complex than what can be characterized with faces alone, applying to all kinds of objects in a meaningful way (35, 36). The assumption that we make with this approach is that the stimulus set of 700 images is rich enough to differentiate between distinct complex neural tuning profiles, even if that tuning is too complex to be human interpretable.

**Fig. 2.**
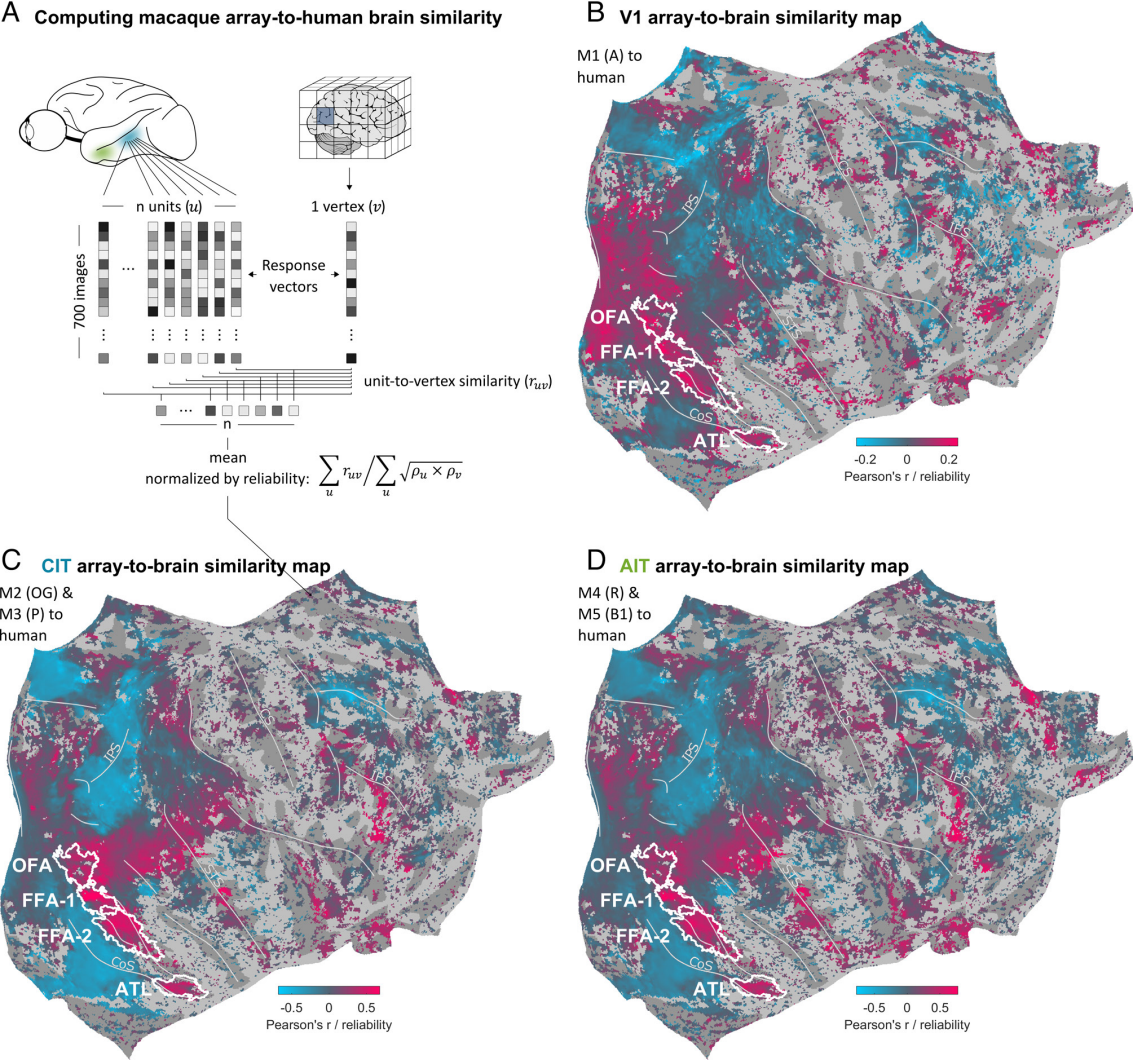
Macaque array-to-human brain similarity maps. (*A*) Illustration of how we computed the similarity between array unit responses and human fMRI vertex responses (subject-averaged in fsaverage space). For each vertex v, the unit-to-vertex correlations $r_{\mathrm{uv}}$ were averaged across units u and then normalized by the Spearman–Brown–corrected joined reliability values $\sqrt{\rho_{u}\times \rho_{v}}$, averaged across units u. (*B*–*D*) Full right hemisphere human brain maps of similarities with macaque V1 array responses (*B*), CIT array responses (*C*), and AIT array responses (*D*). Vertices for which the noise ceiling (unit-averaged joint unit-vertex reliability) was below 0.2 were masked out. White outlines indicate clusters of vertices for which the fraction of subjects that has a given ROI present exceeds 0.33.

We computed the similarity of responses at each specific array location (i.e., V1, CIT, or AIT) to the responses of each vertex of a subject-averaged human brain (Fig. 2*A*; see *Array-to-fMRI Similarity*). Briefly, for a given vertex in the human brain, we took the trial-then-subject-averaged response vector and calculated the Pearson correlation with each individual unit’s trial-averaged response vector. We also calculated a joint reliability for each unit-to-vertex combination, based on the unit’s trial-wise split-half reliability and the vertex’s subject-wise split half reliability. The macaque array-to-human brain similarity was then computed by averaging for each vertex the Pearson correlations separately across V1, CIT, and AIT units, and normalizing by the noise ceiling given by the unit-averaged joint reliability values.

This analysis yielded three macaque array-to-human brain similarity maps, based on the right human hemisphere: one for V1 units (Fig. 2*B*), one for CIT units (Fig. 2*C*), and one for AIT units (Fig. 2*D*). The array-to-brain similarity maps showed a smooth, graded range of negative to positive correlation values, extending well beyond visual areas. This is consistent with a widespread, correlated engagement of the cortex in response to these images. The map based on the macaque V1 array shows similarity biased toward earlier visual areas, although it also shows marked similarity with the higher human face areas. The maps based on macaque CIT and AIT arrays, in contrast, are biased toward higher human visual areas, particularly face regions, as well as regions beyond the visual cortex. The perceptual difference between maps based on CIT and AIT was less clear and required a more direct comparison.

**Fig. 3.**
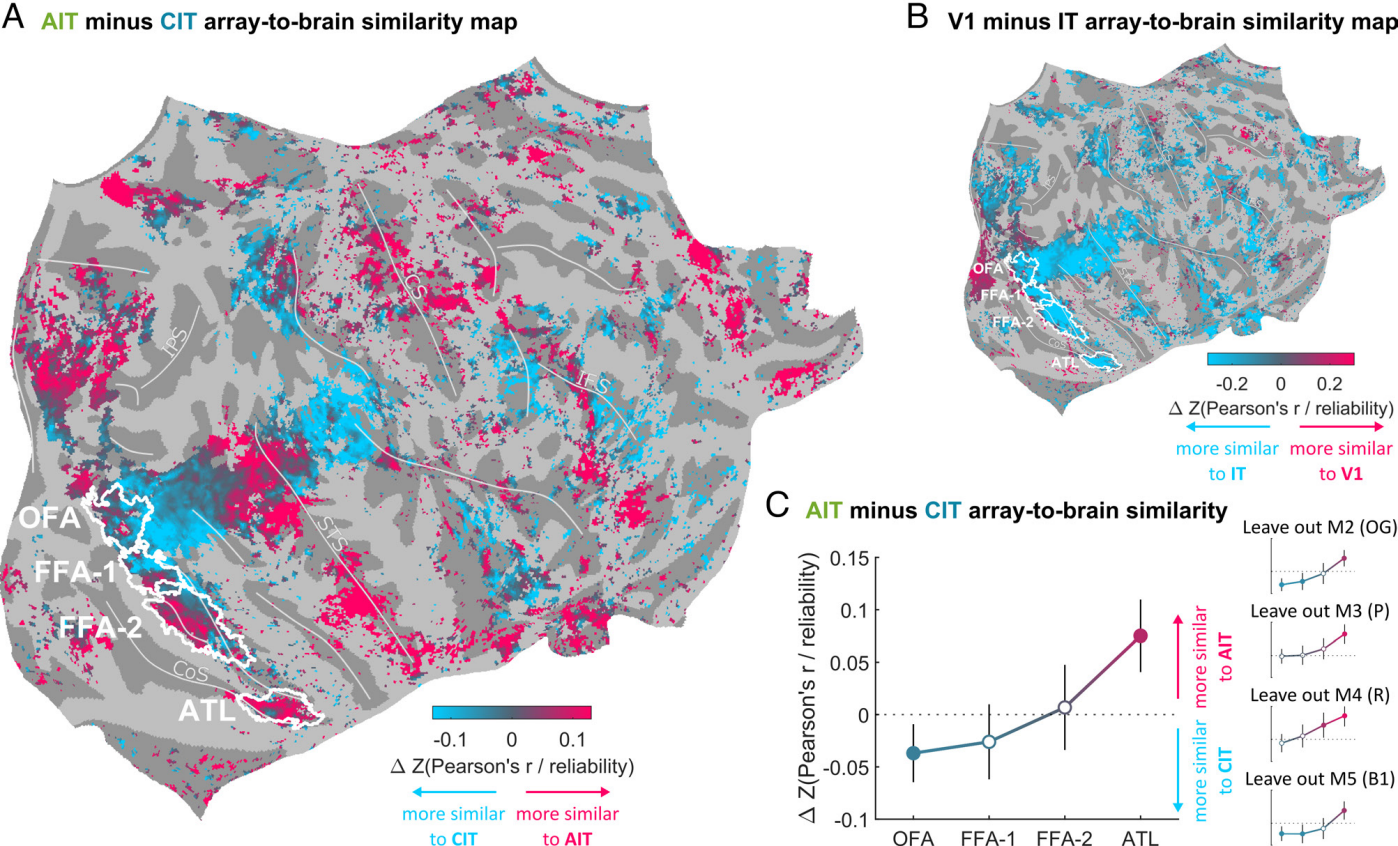
Monkey AIT matched higher human visual (face) areas better than did monkey CIT. (*A*) Full right hemisphere human brain map showing the difference in similarity with macaque AIT and CIT array responses (subject-averaged in fsaverage space). Red hues indicate higher similarity to AIT units than to CIT units. Blue hues indicate higher similarity to CIT units than to AIT units. (*B*) Human brain map showing the difference in similarity with macaque V1 and IT array responses (red hues: higher similarity to AIT units; blue hues: higher similarity to V1). (*C*) AIT minus CIT array-to-brain similarity values averaged for the ROI contours indicated in (*A*). Error bars indicate 95% CIs based on a two-sample *t* test comparing AIT units (N = 116) to CIT units (N = 48). Filled markers denote *P* < 0.05. Insets show the consistency of this trend across monkeys by repeating the analysis while systematically leaving out one monkey at a time (akin to jackknife resampling).

To better assess these differences, we Fisher Z-transformed the maps (see *Fisher Z-Transformation*) and subtracted the map based on CIT from the map based on AIT (Fig. 3*A*). We further masked out vertices that were not correlated or were anticorrelated with both AIT and CIT (Pearson’s *r*/reliability < 0.1). In this AIT minus CIT map, red hues indicate vertices in the human brain that responded more like AIT units than like CIT units. Blue hues indicate vertices that responded more like CIT. This visualization more clearly highlights the fine-grained differences between response similarities with AIT and with CIT. As a sanity check, we subtracted a map based on all IT units from a map based on V1 units (Fig. 3*B*). This V1 minus IT map confirmed that vertices in the early visual cortex responded more like V1 units (red hues), whereas vertices in the higher visual cortex and beyond responded more like IT units (blue hues).

Visual inspection of the response similarities with the target human face regions indicates that human OFA and FFA-1 responses were more like macaque CIT than AIT (predominantly blue hues), whereas human ATL responses were more like macaque AIT than CIT (predominantly red hues). This was less clear for FFA-2: Some vertices corresponded better to CIT, others to AIT. For a quantitative analysis, we averaged unit-to-vertex correlations across vertices for which the fraction of subjects that had a given ROI present exceeded 0.33. This analysis confirmed the posterior-to-anterior trend observed through visual inspection, culminating in the largest AIT minus CIT difference in ATL (Fig. 3*C*).

Since FFA vertices did not show a higher similarity to AIT than to CIT units, these results are inconsistent with scenario 1, where the macaque AIT face region AL corresponds to human FFA (and CIT face region ML to human OFA). However, thus far, these results remain consistent with both scenarios 2 and 3. Next, we used each human subject’s individually localized ROIs in both the left and right hemispheres to determine whether macaque AIT units are better aligned with human FFA-2 (scenario 2) or with ATL (scenario 3).

### Macaque CIT Maps Best Onto Human FFA, and AIT Onto ATL.

**Fig. 4.**
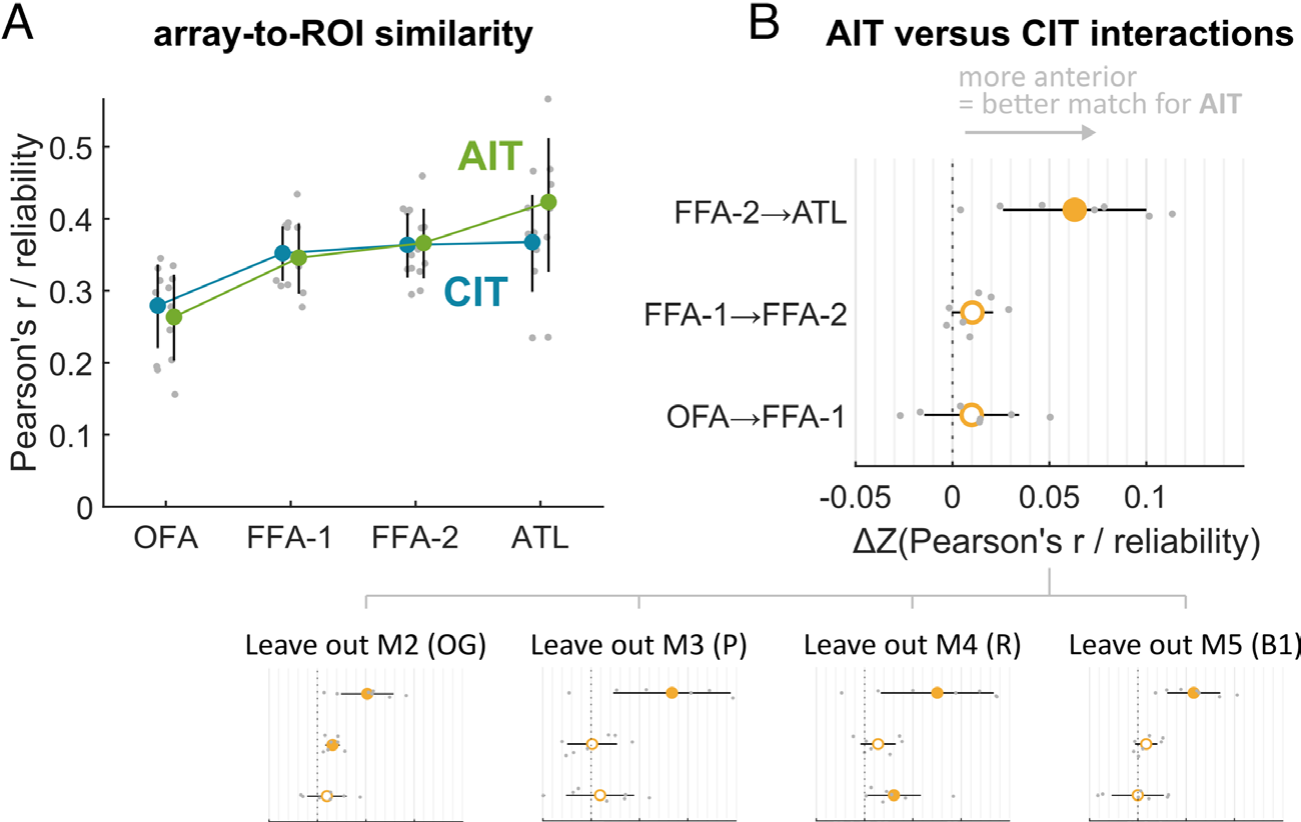
Monkey AIT matched ATL better than it matched FFA. (*A*) Average array-to-ROI similarity. Gray markers represent individual human subjects. Error bars indicate 95% CIs based on a one-sample *t* test (N = 7 human subjects). (*B*) Regression interaction terms between monkey array (AIT vs. CIT) and a one-step transition in the human ROI cascade (ATL vs. FFA-2, FFA-2 vs. FFA-1, or FFA-1 vs. OFA). For example, for the interaction with ATL vs. FFA-2 (indicated as FFA-2 -> ATL in the figure), the positive value means that the increase in array-to-brain similarity from human FFA-2 to ATL is larger for macaque AIT than for CIT. Error bars indicate 95% CIs based on a paired-sample *t* test (N = 7 human subjects). Filled markers denote *P* < 0.05. Insets show the consistency of this trend across monkeys by repeating the analysis while systematically leaving out one monkey at a time (akin to jackknife resampling).

To further arbitrate between scenario 2 and 3 and to account for human subject-level variability, we computed the similarity of monkey array units to each human subject’s individually defined ROIs. The methods to compute monkey array-to-human brain similarity were analogous to Fig. 2*A* but based on the fMRI voxel responses (trial-averaged beta values) of each human subject’s native space. For each human subject, we then averaged array-to-brain similarity values across all voxels within each ROI (Fig. 4*A*).

For both CIT arrays and AIT arrays, there was no significant difference in similarity to human brain responses in FFA-2 versus FFA-1 [CIT: t(6) = 0.61, *P* = 0.5639; AIT: t(6) =1 .11, *P* = 0.3094]. There was also no statistically significant interaction between AIT versus CIT arrays and the difference in similarity to human brain responses in FFA-2 versus FFA-1 [t(6) = 2.35, *P* = 0.0571; see Fig. 4*A*]. These results are inconsistent with the notion that macaque ML and AL are best distinguished in how well they match human FFA-1 and FFA-2 (scenario 2).

In contrast, there was a significant interaction between AIT versus CIT arrays and the difference in similarity to human brain responses in ATL versus FFA-2 [t(6) = 4.17, *P* = 0.0059]. Indeed, when pooling similarity to FFA-1 and FFA-2, we found for AIT arrays that similarity to ATL was significantly higher than similarity to FFA [t(6) = 2.71, *P* = 0.0353] but not for CIT arrays [t(6) = 0.55, *P* = 0.6010]. Similarity to OFA was significantly lower than similarity to FFA for both AIT arrays [t(6) = 3.81, *P* = 0.0089] and CIT arrays [t(6) = 2.66, *P* = 0.0374] confirming the rejection of scenario 1. Thus, overall, these results support the notion that macaque AL is functionally best aligned to human ATL, and macaque ML to human FFA (scenario 3).

## Discussion

We introduce a general framework for identifying fine-grained functional correspondence across species by leveraging population-level neural responses to naturalistic stimuli. Applying this approach to the ventral face patch system, we demonstrate that rich, graded response patterns provide a robust and scalable basis for mapping functional correspondence across species and measurement modalities, without requiring predefined hypotheses or hand-picked stimuli. By directly comparing macaque electrophysiology with human fMRI responses to a shared set of natural scenes, we identified a functional alignment of face-selective areas in which macaque ML corresponds to human FFA and AL to ATL. Despite being derived purely from neural responses, this mapping was consistent with predictions from anatomical topology, suggesting that large-scale cortical layout remains a strong predictor of functional organization across primates. These findings illustrate that neural responses to natural scenes can serve as a reliable basis for cross-species functional inference, offering a flexible, hypothesis-agnostic alternative to traditional methods for comparative neuroscience.

These results help resolve a long-standing ambiguity in the posterior-to-anterior cross-species alignment of face-selective regions. Previous accounts have proposed three competing scenarios: 1) ML aligns with OFA and AL with FFA; 2) ML aligns with FFA-1 and AL with FFA-2; 3) ML aligns with FFA and AL with ATL. Using response patterns across a large set of natural images, we found that recordings from macaque CIT (targeting ML) correlated best with human FFA (both posterior and anterior), with no additional increase for ATL, thereby rejecting scenario 1. In contrast, recordings from macaque AIT (targeting AL) correlated most strongly with human ATL, ruling out scenario 2. Finally, our analyses showed that ML and AL are best distinguished by their correspondence to anterior FFA versus ATL. Together, these findings support scenario 3: a functional alignment of ML with FFA and AL with ATL. This alignment suggests a more anterior human homologue of AL than is often assumed, underscoring the importance of considering broad representational properties in the higher-order cortex. It is worth noting, however, that ATL was the only anterior face-selective region reliably localized across human participants. While our results support an AL-to-ATL correspondence, we cannot rule out the possibility that some of our AIT sites would have aligned more closely with other anterior human regions had those regions been detectable.

One potential explanation for previous discrepancies based on cortical topography (10, 41, 42) is that evolutionary changes may reshape cortical organization (43–45). Such changes can undermine the reliability of using local topography to infer functional correspondence. On the other hand, our conclusions do converge with a posterior-to-anterior mapping derived from full-brain anatomical warping (39), suggesting that more holistic anatomical approaches may better capture conserved organizational principles. Previous studies have also compared a range of functional properties, such as mirror-symmetric tuning, the face inversion effect, face familiarity, and face selectivity, often using small sets of diagnostic stimuli (8–12, 53–55). While these approaches are attractive for their simplicity and interpretability, they are constrained by their reliance on small and highly curated stimulus sets and may miss broader feature tuning. Moreover, recent work has suggested that observations of mirror-symmetric tuning in the human face selective areas may originate from low-level confounds and analysis choices (47, 48). Importantly, these observations highlight a common theme: broader approaches, whether leveraging full-brain anatomical alignment or a comprehensive range of natural stimuli, converge on a consistent cross-species mapping, whereas narrower methods based on local topography or curated stimuli can yield divergent conclusions (56).

More broadly, our findings illustrate how data-driven approaches using naturalistic stimuli offer a powerful tool for fine-grained functional alignment well beyond the domain of face-selective regions. Traditional methods that rely on small, highly curated stimulus sets risk drawing inaccurate conclusions when the chosen stimuli fail to capture the richness of underlying neural representations. Under stimulus-poor conditions, the inclusion or exclusion of a single image can drastically impact observed response profiles. For example, while face-selective regions in both species responded more strongly to faces on average, the specific images that elicited the strongest responses (Fig. 1, “best” images) differed across species—highlighting nuanced differences in feature tuning that could be either amplified or overlooked with only a few stimuli. These challenges are exacerbated when the stimuli are overly constrained by a conceptual notion such as discrete stimulus categories (57). Growing evidence suggests that the high-level visual cortex, including face-selective cells, is best characterized by an integrated feature space, where category-selective responses are carried by domain-general features (35, 36, 58–61). By sampling broadly from the natural image space, our approach captures these representational subtleties without imposing categorical assumptions. This strategy enables a more flexible, scalable, and hypothesis-agnostic framework for assessing functional alignment, particularly in higher-order areas where feature tuning is complex or poorly understood.

Our approach resonates with perspectives from ecological vision, which argue that perception is attuned to natural environments rather than artificially constrained laboratory stimuli (62–65). This reinforces the value of using naturalistic images to probe representational similarity across species. That said, the images in our study were likely more biased toward human environments, at least at a higher level. Our 700 images were randomly drawn (via a ≥2 repetition constraint unrelated to content) from a 1,000-image subset that was itself randomly sampled from the full, human-centric NSD corpus (50). As such, the stimulus set is likely representative of the full NSD. However, it likely does not reflect the ecological niches of macaques, especially those raised in laboratory settings. In addition, it remains an open question whether natural scenes carry equivalent perceptual meaning across species. Some images may carry different semantic or affective significance for humans than for laboratory-raised macaques, introducing potential nonvisual differences. This would complicate our approach for representations that contain learned semantic or abstract associations (57, 66). Despite these limitations in representativeness, the robust cross-species alignment observed across both early and higher-order ventral visual areas suggests a shared visual representation driven by common image-based properties (13), supporting the use of static natural scenes for cross-species inference even in the absence of matched representativeness or behavioral significance.

Establishing cross-species functional correspondence in primates is a long-standing challenge, complicated by issues such as differences in measurement techniques (7). The advent of fMRI, usable in both monkeys and humans, has enabled an explosion of comparative studies inferring correspondence by comparing fMRI maps of individual functional properties or stimulus contrasts (7–10, 12, 16, 39, 41, 43, 44, 67–72). Our approach demonstrates that direct comparisons between electrophysiological and fMRI data can contribute meaningfully to this line of research. By leveraging population-level neural responses and correlating them with human fMRI responses at each vertex, we were able to delineate functional alignment without requiring parallel imaging experiments in both species. Although the physiological differences between spiking and BOLD signals remain, our results show that image-level tuning in multiunit activity aligns meaningfully with human fMRI responses (73–75). Indeed, the array-to-brain correlation maps show the highest similarity in regions consistent with each array’s location: human early visual cortex for the macaque V1 array and ventral temporal cortex (near OFA, FFA, and ATL) for the IT arrays. Furthermore, our results converge with previously hypothesized homologies derived from anatomical warping of fMRI-localized macaque face patches onto a human flat map (39, 40). Overall, the fact that our framework captured meaningful alignment across both coarse and fine levels of cortical organization – from early versus high-level visual areas to distinctions among neighboring face regions – suggests that this approach can generalize to systems beyond the ventral stream, wherever shared representations exist across species.

While our results clarify the alignment of ML with FFA and suggest an alignment of AL with ATL, the broader correspondence of face-selective regions remains less well understood. By posterior extrapolation, our findings are consistent with a PL-OFA correspondence (9, 41, 76, 77), though we did not test this directly. At the anterior end, identifying human homologs for macaque AM, the anterior fundus patch (AF), and temporal pole patch (TP) (55), remains particularly challenging. Although Rajimehr et al. (39) projected an anterior temporal face patch (“ATFP”) onto the human cortex, they did not further subdivide this region due to inconsistent detectability, even though it encompassed territories consistent with AL, AF, and AM as originally defined by Tsao et al. (8, 78). In general, such anterior macaque face regions may correspond to multiple human anterior temporal face areas, including medial sites such as the perirhinal cortex and temporal pole (54, 55). However, signal dropout, variability in patch detectability, conflation of nearby regions, and differences in analysis choices all complicate the definition of clear one-to-one correspondences in this region (39, 77, 79–82). Indeed, in our NSD analyses, we excluded a medial temporal face area because it was localized in only two participants. Other face-selective regions, such as the macaque middle dorsal (MD), middle fundus (MF), and anterior fundus (AF) patches, and the human posterior superior temporal sulcus (pSTS), are more reliably localized using movies of facial motion (76, 83). Given this complexity, future work incorporating denser sampling, improved imaging, dynamic stimuli, and relative anatomical location will be essential for resolving the full correspondence of the primate face patch system.

Moving forward, several directions could extend the present work. First, because we recorded from only one array per monkey, interindividual differences are confounded with between-area differences. While leave-one-subject-out analyses confirmed that no single monkey drove the observed effects, within-subject sampling will be important for resolving finer distinctions. Second, future work should expand this approach beyond the face network to other parts of the inferotemporal cortex or other visual and cognitive areas (17), potentially leveraging full-brain monkey fMRI to support functional warping. Extending into these territories will likely require more complex paradigms that further enhance ecological and behavioral richness, such as dynamic stimuli, active viewing, and higher-order dimensions like value, learning, or semantic associations. While nontrivial in nonhuman species, incorporating behavioral context and task demands could help disentangle bottom–up from top–down influences and clarify how attention, recurrent processing, and internal states modulate shared representations. Finally, to establish phylogenetic homology in the strict evolutionary sense, the functional alignment demonstrated here will need to be complemented by converging evidence from cytoarchitecture, connectivity, and developmental lineage, which is often inaccessible in humans or scarce for functionally defined brain regions (84). In this context, our approach provides precise functional targets that can guide future investigations into whether putatively corresponding areas share conserved structural or developmental features. Such integration of structure and function will be critical for grounding the search for homology.

In conclusion, our results point toward a flexible, data-driven framework for establishing functional correspondences across species and modalities. By using naturalistic stimuli to capture rich, high-dimensional, and graded response patterns, this approach overcomes the limitations of traditional alignment methods that rely on narrow stimulus sets or predefined functional heuristics. Although we demonstrated this in the ventral visual face patch system, this method is broadly applicable, offering a general framework for establishing cross-species alignment in domains where naturalistic data can be collected. As technological advances continue to enable more large-scale and brain-wide recordings in nonhuman primates (17), frameworks that scale with this complexity and support cross-species comparisons of increasingly abstract, hard-to-define functions will be essential. Our findings highlight the value of naturalistic, data-driven alignment as a foundation for such efforts, offering both a principled path toward unified models of brain function and a powerful complement to anatomical and developmental approaches in the study of brain evolution.

## Materials and Methods

### NSD Data.

The human data analyzed in this study were obtained from the NSD, a large-scale fMRI dataset comprising whole-brain, high-resolution measurements collected from eight subjects who viewed natural scenes while engaged in a continuous recognition task. Details regarding data collection and preprocessing can be found in the original publication (50). Face-selective regions were defined based on the results of the category-selective functional localizer included in the NSD experiments. Labels for these regions were assigned by the NSD authors. For the subject-averaged human brain maps, the provided probabilistic localizer maps were used with a threshold of 0.33 (i.e., only vertices corresponding to a face-selective region in at least one-third of the subjects were included). We additionally defined each subject’s face-selective ROIs individually, based on their native-space face localizer t-maps, using a threshold of t > 3. One subject was excluded because no anterior face region could be defined based on the functional localizer, resulting in a final sample of seven subjects. In addition, a medial temporal lobe face area that could be defined in only two remaining subjects was excluded.

### Animals and Arrays.

Five adult male macaques were used in this experiment: four rhesus macaques (*Macaca mulatta*; initials A, OG, P, and B1—referred to as M1, M2, M3, and M5, respectively) aged 8 to 16 y and one pigtailed macaque (*Macaca nemestrina*; initial R, referred to as M4), aged 13 y. All five monkeys were implanted with chronic microelectrode arrays: one in V1 and four in the lower bank of the superior temporal sulcus. Specifically, two monkeys were implanted with 32-channel floating microelectrode arrays (FMA; Microprobes for Life Sciences, Gaithersburg, MD): V1 of M1 and CIT of M3. Three monkeys were implanted with 64-channel NiCr microwire bundle arrays (Microprobes for Life Sciences, Gaithersburg, MD) (85): CIT of M2 and AIT of M4 and M5. The target location for the face patch arrays was identified using fMRI face localizers (M3, M4, M5) or anatomical landmarks [M2; STS “bumps:” Arcaro et al. (86)]. All procedures were approved by the Harvard Medical School Institutional Animal Care and Use Committee and conformed to NIH guidelines provided in the Guide for the Care and Use of Laboratory Animals.

### Experiments.

During recordings, the monkeys were performing a fixation task in which they were rewarded with drops of juice to fix their gaze on a spot in the middle of a 53-cm LCD monitor. The gaze position was monitored using an ISCAN system (ISCAN, Woburn, MA). The experiments were controlled with MonkeyLogic (https://monkeylogic.nimh.nih.gov/). During fixation, images were presented at a size of 8 visual degrees and a rate of 100 ms on and 100 ms off. The images were presented at the center of the mapped receptive field, with a jitter of ±1 to ±2 visual degrees for IT arrays. An average of 32 trials were presented per stimulus.

### Stimuli.

The stimulus set consisted of a subset of 1,000 images that were shared across human subjects in the NSD (50). The NSD image set contains a rich variety of photographs taken from Microsoft’s Common Objects in Context [MS COCO (87)] image database. For this paper, we focus on 700 images for which there were at least two presentations for each human subject, to be able to compute a split-half reliability using the exact same images for each subject.

fMRI-guided array targeting

The target location for the array placement for 3 out of 4 monkeys (M3, M4, M5) was identified using an fMRI face localizer. The details of the fMRI experiments are described in Arcaro and Livingstone (88) and will only be summarized here briefly. The monkeys were scanned using custom-made four-channel surface coils (by A. Maryam at the Martinos Imaging Center), in a 3 T TIM Trio scanner with an AC88 gradient insert. Functional images were acquired using a repetition time = 2 s, echo time = 13 ms, flip angle = 72°, iPAT = 2, matrix size = 96 × 96 mm, resolution = 1-mm isotropic, and 67 contiguous sagittal slices. To enhance signal-to-noise ratio and increase contrast (89), the monkeys were injected with 12 mg/kg of monocrystalline iron oxide nanoparticles (Feraheme, AMAG Pharmaceuticals, Cambridge, MA, USA) before each scanning session. The face localizer consisted of randomly shuffled 20 s blocks of face or nonface objects, interleaved with 20 s of a neutral gray screen.

### Data Analysis.

#### Firing rates.

To compute average firing rates, we counted the number of spikes in a 200 ms window following stimulus presentation onset. The latency of the response was selected based on a visual assessment of the global average (across channels and stimuli) peristimulus time histogram, ranging from 65 ms to 125 ms. Following previous procedures (35, 36), we used an a priori response reliability criterion of >0.4 to include only visually driven, selective neural units for further analysis. This yielded 11 multiunit sites from V1 recordings, 48 multiunit sites from CIT recordings, and 116 multiunit sites from AIT recordings.

#### Response reliability.

We determined a trial-wise split-half reliability per neural unit and per voxel in each NSD subject brain, and we determined a subject-wise split-half reliability per vertex in the NSD “fsaverage” space. To obtain trial-wise split-half reliability, we first computed the correlation r between the average response vector based on odd trials and the one based on even trials. We then applied the Spearman–Brown correction to obtain a reliability $\rho =2r/\left(1+r\right)$.

To obtain subject-wise split-half reliability, r was computed as the correlation obtained from the trial-and-subject averaged response vectors for every possible way to split the NSD subjects in two, averaged across splits.

#### Face selectivity.

We assessed face selectivity by computing the face versus no-face d′ sensitivity index comparing trial-averaged responses to a subset of images with prominent primate faces (N = 26 out of all 700 images) versus all images without faces or animals (N = 255 out of all 700 images):

$$d^{\prime }=\frac{\mu_{F}-\mu_{\mathrm{NF}}}{\sqrt{(\sigma_{F}^{2}+\sigma_{\mathrm{NF}}^{2})/2}},$$

where $\mu_{F}$ and $\mu_{\mathrm{NF}}$ are the across-stimulus means for faces and nonfaces, and $\sigma_{F}$ and $\sigma_{\mathrm{NF}}$ are the across-stimulus SDs.

#### Array-to-fMRI similarity.

We quantified the similarity between microelectrode array responses and fMRI vertex/voxel responses as follows. For each fMRI vertex/voxel v and array multiunit u, we computed the Pearson correlation $r_{\mathrm{uv}}$ between their respective response vectors across images. For the flat maps, we then averaged the unit-to-vertex correlation values across all array units u to obtain a single correlation value for each vertex v. For the ROIs, we averaged across all units and voxels to obtain a single correlation value for each ROI.

To estimate a noise ceiling, we computed joint reliability values as $\sqrt{\rho_{u}\times \rho_{v}}$, where $\rho_{u}$ and $\rho_{v}$ represent the response reliability of each unit and each vertex/voxel, respectively (see *Response Reliability*). Similar to the correlation values, we averaged these joint reliability values across units (and voxels in the case of ROIs) to obtain a single noise ceiling value for each vertex v or each ROI.

Finally, we normalized each array-to-vertex/ROI correlation by its corresponding noise ceiling to obtain a single array-to-vertex/ROI similarity value.

#### Fisher Z-transformation.

The correlation-based array-to-fMRI similarity values are inherently bounded between -1 and 1 and exhibit a skewed distribution, especially near the extremes. Therefore, for statistical comparison between similarity values associated with a given vertex/ROI, we applied the Fisher Z-transformation, $Z\left(r\right)=atanh\left(r\right)$, to normalize the distribution and stabilize variance across the range of similarity values (90).

## Data Availability

All data needed to reproduce the main results are publicly available. NSD data are shared by the original authors at https://naturalscenesdataset.org/ (51). The macaque neural data are shared on the open science framework at https://osf.io/vh2pd/ (52).

## References

[1] R. Malach , Object-related activity revealed by functional magnetic resonance imaging in human occipital cortex. Proc. Natl. Acad. Sci. **92**, 8135–8139 (1995). 7667258 10.1073/pnas.92.18.8135 PMC41110

[2] Z. Kourtzi, N. Kanwisher, Cortical regions involved in perceiving object shape. J. Neurosci. **20**, 3310–3318 (2000). 10777794 10.1523/JNEUROSCI.20-09-03310.2000 PMC6773111

[3] N. Kanwisher, J. McDermott, M. M. Chun, The fusiform face area: A module in human extrastriate cortex specialized for face perception. J. Neurosci. **17**, 4302–4311 (1997). 9151747 10.1523/JNEUROSCI.17-11-04302.1997 PMC6573547

[4] P. E. Downing, Y. Jiang, M. Shuman, N. Kanwisher, A cortical area selective for visual processing of the human body. Science **1979**, 2470–2473 (2001). 10.1126/science.1063414 11577239

[5] K. Grill-Spector , A sequence of object-processing stages revealed by fMRI in the human occipital lobe. Hum. Brain Mapp. **6**, 316–328 (1998). 9704268 10.1002/(SICI)1097-0193(1998)6:4<316::AID-HBM9>3.0.CO;2-6 PMC6873387

[6] J. Vinberg, K. Grill-Spector, Representation of shapes, edges, and surfaces across multiple cues in the human visual cortex. J. Neurophysiol. **99**, 1380–1393 (2008). 18171705 10.1152/jn.01223.2007

[7] M. I. Sereno, R. B. Tootell, From monkeys to humans: What do we now know about brain homologies? Curr. Opin. Neurobiol. **15**, 135–144 (2005). 15831394 10.1016/j.conb.2005.03.014

[8] D. Y. Tsao, S. Moeller, W. A. Freiwald, Comparing face patch systems in macaques and humans. Proc. Natl. Acad. Sci. **105**, 19514–19519 (2008). 19033466 10.1073/pnas.0809662105 PMC2614792

[9] G. Yovel, W. A. Freiwald, Face recognition systems in monkey and human: Are they the same thing? F1000Prime Rep. **5**, 10 (2013). 23585928 10.12703/P5-10 PMC3619156

[10] M. A. Pinsk , Neural representations of faces and body parts in macaque and human cortex: A comparative fMRI study. J. Neurophysiol. **101**, 2581–2600 (2009). 19225169 10.1152/jn.91198.2008 PMC2681436

[11] J. Dubois, A. O. de Berker, D. Y. Tsao, Single-unit recordings in the macaque face patch system reveal limitations of fMRI MVPA. J. Neurosci. **35**, 2791–2802 (2015). 25673866 10.1523/JNEUROSCI.4037-14.2015 PMC4323541

[12] A. H. Bell, F. Hadj-Bouziane, J. B. Frihauf, R. B. H. Tootell, L. G. Ungerleider, Object representations in the temporal cortex of monkeys and humans as revealed by functional magnetic resonance imaging. J. Neurophysiol. **101**, 688–700 (2009). 19052111 10.1152/jn.90657.2008 PMC2657058

[13] N. Kriegeskorte , Matching categorical object representations in inferior temporal cortex of man and monkey. Neuron **60**, 1126–1141 (2008). 19109916 10.1016/j.neuron.2008.10.043 PMC3143574

[14] M. Carandini , Do we know what the early visual system does?. J. Neurosci. **25**, 10577–10597 (2005). 16291931 10.1523/JNEUROSCI.3726-05.2005 PMC6725861

[15] G. Felsen, J. Touryan, F. Han, Y. Dan, Cortical sensitivity to visual features in natural scenes. PLoS Biol. **3**, e342 (2005). 16171408 10.1371/journal.pbio.0030342 PMC1233414

[16] R. B. H. Tootell, D. Tsao, W. Vanduffel, Neuroimaging weighs in: Humans meet macaques in “primate” visual cortex. J. Neurosci. **23**, 3981–3989 (2003). 12764082 10.1523/JNEUROSCI.23-10-03981.2003 PMC6741079

[17] E. M. Trautmann , Large-scale high-density brain-wide neural recording in nonhuman primates. Nat. Neurosci. **28**, 1562–1575 (2025). 40551025 10.1038/s41593-025-01976-5 PMC12229894

[18] D. Y. Tsao, W. A. Freiwald, R. B. H. Tootell, M. S. Livingstone, A cortical region consisting entirely of face-selective cells. Science **311**, 670–674 (2006). 16456083 10.1126/science.1119983 PMC2678572

[19] W. A. Freiwald, D. Y. Tsao, M. S. Livingstone, A face feature space in the macaque temporal lobe. Nat. Neurosci. **12**, 1187–1196 (2009). 19668199 10.1038/nn.2363 PMC2819705

[20] S. Moeller, T. Crapse, L. Chang, D. Y. Tsao, The effect of face patch microstimulation on perception of faces and objects. Nat. Neurosci. **20**, 743–752 (2017). 28288127 10.1038/nn.4527 PMC8086516

[21] P. Grimaldi, K. S. Saleem, D. Tsao, Anatomical Connections of the Functionally Defined “Face Patches” in the Macaque Monkey. Neuron **90**, 1325–1342 (2016). 27263973 10.1016/j.neuron.2016.05.009 PMC5573145

[22] W. A. Freiwald, D. Y. Tsao, Functional compartmentalization and viewpoint generalization within the macaque face-processing system. Science **1979**, 845–851 (2010). 10.1126/science.1194908 PMC3181095 21051642

[23] L. Chang, D. Y. Tsao, The code for facial identity in the primate brain. Cell **169**, 1013–1028.e14 (2017). 28575666 10.1016/j.cell.2017.05.011 PMC8088389

[24] S. Sadagopan, W. Zarco, W. A. Freiwald, A causal relationship between face-patch activity and face-detection behavior. eLife **6**, e18558 (2017). 28375078 10.7554/eLife.18558 PMC5380432

[25] R. Azadi, E. Lopez, J. Taubert, A. Patterson, A. Afraz, Inactivation of face-selective neurons alters eye movements when free viewing faces. Proc. Natl. Acad. Sci. **121**, e2309906121 (2024). 38198528 10.1073/pnas.2309906121 PMC10801883

[26] S.-R. Afraz, R. Kiani, H. Esteky, Microstimulation of inferotemporal cortex influences face categorization. Nature **442**, 692–695 (2006). 16878143 10.1038/nature04982

[27] A. Afraz, E. S. Boyden, J. J. DiCarlo, Optogenetic and pharmacological suppression of spatial clusters of face neurons reveal their causal role in face gender discrimination. Proc. Natl. Acad. Sci. U.S.A. **112**, 6730–6735 (2015). 25953336 10.1073/pnas.1423328112 PMC4450412

[28] E. N. Waidmann, K. W. Koyano, J. J. Hong, B. E. Russ, D. A. Leopold, Local features drive identity responses in macaque anterior face patches. Nat. Commun. **13**, 5592 (2022). 36151142 10.1038/s41467-022-33240-w PMC9508131

[29] A. P. Khandhadia, A. P. Murphy, L. M. Romanski, J. K. Bizley, D. A. Leopold, Audiovisual integration in macaque face patch neurons. Curr. Biol. **31**, 1826–1835.e3 (2021). 33636119 10.1016/j.cub.2021.01.102 PMC8521527

[30] E. Premereur, J. Taubert, P. Janssen, R. Vogels, W. Vanduffel, Effective connectivity reveals largely independent parallel networks of face and body patches. Curr. Biol. **26**, 3269–3279 (2016). 27866893 10.1016/j.cub.2016.09.059

[31] J. Taubert, G. Van Belle, W. Vanduffel, B. Rossion, R. Vogels, The effect of face inversion for neurons inside and outside fMRI-defined face-selective cortical regions. J. Neurophysiol. **113**, 1644–1655 (2015). 25520434 10.1152/jn.00700.2014 PMC4346728

[32] J. Taubert, V. Goffaux, G. Van Belle, W. Vanduffel, R. Vogels, The impact of orientation filtering on face-selective neurons in monkey inferior temporal cortex. Sci. Rep. **6**, 21189 (2016). 26879148 10.1038/srep21189 PMC4754760

[33] A. Bardon, W. Xiao, C. R. Ponce, M. S. Livingstone, G. Kreiman, Face neurons encode nonsemantic features. Proc. Natl. Acad. Sci. **119**, e2118705119 (2022). 35377737 10.1073/pnas.2118705119 PMC9169805

[34] C. R. Ponce , Evolving images for visual neurons using a deep generative network reveals coding principles and neuronal preferences. Cell **177**, 999–1009.e10 (2019). 31051108 10.1016/j.cell.2019.04.005 PMC6718199

[35] S. Sharma, K. Vinken, A. V. Jagadeesh, M. S. Livingstone, Face cells encode object parts more than facial configuration of illusory faces. Nat. Commun. **15**, 9879 (2024). 39543127 10.1038/s41467-024-54323-w PMC11564726

[36] K. Vinken, J. S. Prince, T. Konkle, M. S. Livingstone, The neural code for “face cells” is not face-specific. Sci. Adv. **9**, eadg1736 (2023). 37647400 10.1126/sciadv.adg1736 PMC10468123

[37] P. L. Aparicio, E. Issa, J. J. DiCarlo, Neurophysiological organization of the middle face patch in macaque inferior temporal cortex. J. Neurosci. **36**, 12729–12745 (2016). 27810930 10.1523/JNEUROSCI.0237-16.2016 PMC5157113

[38] K. Vinken, R. Vogels, A behavioral face preference deficit in a monkey with an incomplete face patch system. Neuroimage **189**, 415–424 (2019). 30665007 10.1016/j.neuroimage.2019.01.043

[39] R. Rajimehr, J. C. Young, R. B. H. Tootell, An anterior temporal face patch in human cortex, predicted by macaque maps. Proc. Natl. Acad. Sci. **106**, 1995–2000 (2009). 19179278 10.1073/pnas.0807304106 PMC2632713

[40] D. Y. Tsao, W. A. Freiwald, T. A. Knutsen, J. B. Mandeville, R. B. H. Tootell, Faces and objects in macaque cerebral cortex. Nat. Neurosci. **6**, 989–995 (2003). 12925854 10.1038/nn1111 PMC8117179

[41] R. Lafer-Sousa, B. R. Conway, N. G. Kanwisher, Color-biased regions of the ventral visual pathway lie between face- and place-selective regions in humans, as in macaques. J. Neurosci. **36**, 1682–1697 (2016). 26843649 10.1523/JNEUROSCI.3164-15.2016 PMC4737777

[42] N. Caspari , Fine-grained stimulus representations in body selective areas of human occipito-temporal cortex. Neuroimage **102**, 484–497 (2014). 25109529 10.1016/j.neuroimage.2014.07.066

[43] G. A. Orban, D. Van Essen, W. Vanduffel, Comparative mapping of higher visual areas in monkeys and humans. Trends Cogn. Sci. **8**, 315–324 (2004). 15242691 10.1016/j.tics.2004.05.009

[44] E. E. Meyer, M. Martynek, S. Kastner, M. S. Livingstone, M. J. Arcaro, Expansion of a conserved architecture drives the evolution of the primate visual cortex. Proc. Natl. Acad. Sci. **122**, e2421585122 (2025). 39805017 10.1073/pnas.2421585122 PMC11761675

[45] A. C. Halley, L. Krubitzer, Not all cortical expansions are the same: The coevolution of the neocortex and the dorsal thalamus in mammals. Curr. Opin. Neurobiol. **56**, 78–86 (2019). 30658218 10.1016/j.conb.2018.12.003 PMC6551301

[46] V. Axelrod, G. Yovel, Hierarchical processing of face viewpoint in human visual cortex. J. Neurosci. **32**, 2442–2452 (2012). 22396418 10.1523/JNEUROSCI.4770-11.2012 PMC6621816

[47] F. M. Ramírez, R. M. Cichy, C. Allefeld, J.-D. Haynes, The neural code for face orientation in the human fusiform face area. J. Neurosci. **34**, 12155–12167 (2014). 25186759 10.1523/JNEUROSCI.3156-13.2014 PMC6608457

[48] C. Revsine, J. Gonzalez-Castillo, E. P. Merriam, P. A. Bandettini, F. M. Ramírez, A unifying model for discordant and concordant results in human neuroimaging studies of facial viewpoint selectivity. J. Neurosci. **44**, e0296232024 (2024). 38438256 10.1523/JNEUROSCI.0296-23.2024 PMC11044116

[49] G. Schalk , Facephenes and rainbows: Causal evidence for functional and anatomical specificity of face and color processing in the human brain. Proc. Natl. Acad. Sci. **114**, 12285–12290 (2017). 29087337 10.1073/pnas.1713447114 PMC5699078

[50] E. J. Allen , A massive 7T fMRI dataset to bridge cognitive neuroscience and artificial intelligence. Nat. Neurosci. **25**, 116–126 (2022). 34916659 10.1038/s41593-021-00962-x

[51] E. J. Allen , Natural scenes dataset from “A massive 7T fMRI dataset to bridge cognitive neuroscience and artificial intelligence.” https://naturalscenesdataset.org/. 10.1038/s41593-021-00962-x 34916659

[52] K. Vinken, S. Sharma, M. S. Livingstone, Data from “Mapping Macaque to Human Cortex with Natural Scene Responses.” OSF. https://osf.io/vh2pd/files/osfstorage. Deposited 23 August 2025. 10.1073/pnas.2512619122 PMC12519150 41026824

[53] V. Axelrod, G. Yovel, The challenge of localizing the anterior temporal face area: A possible solution. Neuroimage **81**, 371–380 (2013). 23684864 10.1016/j.neuroimage.2013.05.015

[54] B. Deen, G. Husain, W. A. Freiwald, A familiar face and person processing area in the human temporal pole. Proc. Natl. Acad. Sci. **121**, e2321346121 (2024). 38954551 10.1073/pnas.2321346121 PMC11252731

[55] S. M. Landi, W. A. Freiwald, Two areas for familiar face recognition in the primate brain. Science **1979**, 591–595 (2017). 10.1126/science.aan1139 PMC5612776 28798130

[56] C. Baker, D. Kravitz, Insights from the evolving model of two cortical visual pathways. J. Cogn. Neurosci. **36**, 2568–2579 (2024). 38820560 10.1162/jocn_a_02192 PMC11602006

[57] J. B. Ritchie, S. G. Wardle, M. Vaziri-Pashkam, D. J. Kravitz, C. I. Baker, Rethinking category-selectivity in human visual cortex. Cogn. Neurosci. 1–28 (2025). 10.1080/17588928.2025.2543890 PMC12458057 40836402

[58] F. R. Doshi, T. Konkle, Cortical topographic motifs emerge in a self-organized map of object space. Sci. Adv. **9**, eade8187 (2023). 37343093 10.1126/sciadv.ade8187 PMC10284546

[59] P. Bao, L. She, M. McGill, D. Y. Tsao, A map of object space in primate inferotemporal cortex. Nature **583**, 103–108 (2020). 32494012 10.1038/s41586-020-2350-5 PMC8088388

[60] A. V. Jagadeesh, J. L. Gardner, Texture-like representation of objects in human visual cortex. Proc. Natl. Acad. Sci. **119**, e2115302119 (2022). 35439063 10.1073/pnas.2115302119 PMC9169962

[61] S. Lugtmeijer, A. M. Sobolewska, E. H. F. de Haan, H. S. Scholte, Visual feature processing in a large stroke cohort: Evidence against modular organization. Brain **148**, 1144–1154 (2025). 39799961 10.1093/brain/awaf009 PMC11969467

[62] W. S. Geisler, Visual perception and the statistical properties of natural scenes. Annu. Rev. Psychol. **59**, 167–192 (2008). 17705683 10.1146/annurev.psych.58.110405.085632

[63] D. Purves, R. B. Lotto, Why we see What we do: An Empirical theory of Vision (Sinauer Associates, 2003).

[64] W. H. Warren, Information is where you find it: Perception as an ecologically well-posed problem. i-Perception **12**, 20416695211000366 (2021). 33815740 10.1177/20416695211000366 PMC7995459

[65] H. S. Scholte, E. H. F. de Haan, Beyond binding: From modular to natural vision. Trends Cogn. Sci. **29**, 505–515 (2025). 40234139 10.1016/j.tics.2025.03.002 PMC12135946

[66] J. S. Bruner, C. C. Goodman, Value and need as organizing factors in perception. J. Abnormal Social Psychol. **42**, 33–44 (1947). 10.1037/h0058484 20285707

[67] N. Kanwisher, Animal models of the human brain: Successes, limitations, and alternatives. Curr. Opin. Neurobiol. **90**, 102969 (2025). 39914250 10.1016/j.conb.2024.102969

[68] W. Vanduffel , Extracting 3D from Motion: Differences in Human and Monkey Intraparietal Cortex. Science **1979**, 413–415 (2002). 10.1126/science.1073574 12376701

[69] D. Y. Tsao , Stereopsis activates V3A and caudal intraparietal areas in macaques and humans. Neuron **39**, 555–568 (2003). 12895427 10.1016/s0896-6273(03)00459-8

[70] G. A. Orban , Similarities and differences in motion processing between the human and macaque brain: Evidence from fMRI. Neuropsychologia **41**, 1757–1768 (2003). 14527539 10.1016/s0028-3932(03)00177-5

[71] G. A. Orban , Mapping the parietal cortex of human and non-human primates. Neuropsychologia **44**, 2647–2667 (2006). 16343560 10.1016/j.neuropsychologia.2005.11.001

[72] Z. Kourtzi, A. S. Tolias, C. F. Altmann, M. Augath, N. K. Logothetis, Integration of local features into global shapes: Monkey and human FMRI studies. Neuron **37**, 333–346 (2003). 12546827 10.1016/s0896-6273(02)01174-1

[73] J. B. M. Goense, N. K. Logothetis, Neurophysiology of the BOLD fMRI signal in awake monkeys. Curr. Biol. **18**, 631–640 (2008). 18439825 10.1016/j.cub.2008.03.054

[74] N. K. Logothetis, B. A. Wandell, Interpreting the bold signal. Annu. Rev. Physiol. **66**, 735–769 (2004). 14977420 10.1146/annurev.physiol.66.082602.092845

[75] N. K. Logothetis, J. Pauls, M. Augath, T. Trinath, A. Oeltermann, Neurophysiological investigation of the basis of the fMRI signal. Nature **412**, 150–157 (2001). 11449264 10.1038/35084005

[76] C. Fisher, W. A. Freiwald, Contrasting specializations for facial motion within the macaque face-processing system. Curr. Biol. **25**, 261–266 (2015). 25578903 10.1016/j.cub.2014.11.038 PMC4302012

[77] J. K. Hesse, D. Y. Tsao, The macaque face patch system: A turtle’s underbelly for the brain. Nat. Rev. Neurosci. **21**, 695–716 (2020). 33144718 10.1038/s41583-020-00393-w

[78] S. Moeller, W. A. Freiwald, D. Y. Tsao, Patches with links: A unified system for processing faces in the macaque temporal lobe. Science **1979**, 1355–1359 (2008). 10.1126/science.1157436 PMC8344042 18535247

[79] T. Janssens, Q. Zhu, I. D. Popivanov, W. Vanduffel, Probabilistic and single-subject retinotopic maps reveal the topographic organization of face patches in the macaque cortex. J. Neurosci. **34**, 10156–10167 (2014). 25080579 10.1523/JNEUROSCI.2914-13.2013 PMC6608270

[80] K. S. Weiner, K. Grill-Spector, The improbable simplicity of the fusiform face area. Trends Cogn. Sci. **16**, 251–254 (2012). 22481071 10.1016/j.tics.2012.03.003

[81] S. Nasr, R. B. H. Tootell, Role of fusiform and anterior temporal cortical areas in facial recognition. Neuroimage **63**, 1743–1753 (2012). 23034518 10.1016/j.neuroimage.2012.08.031 PMC3472036

[82] N. Caspari , Fine-grained stimulus representations in body selective areas of human occipito-temporal cortex. Neuroimage **102**, 484–497 (2014). 25109529 10.1016/j.neuroimage.2014.07.066

[83] K. S. Weiner, K. Grill-Spector, The evolution of face processing networks. Trends Cogn. Sci. **19**, 240–241 (2015). 25840651 10.1016/j.tics.2015.03.010 PMC4414913

[84] P. Grimaldi, K. S. Saleem, D. Tsao, Anatomical connections of the functionally defined “face patches” in the macaque monkey. Neuron **90**, 1325–1342 (2016). 27263973 10.1016/j.neuron.2016.05.009 PMC5573145

[85] D. B. T. McMahon, I. V. Bondar, O. A. T. Afuwape, D. C. Ide, D. A. Leopold, One month in the life of a neuron: Longitudinal single-unit electrophysiology in the monkey visual system. J. Neurophysiol. **112**, 1748–1762 (2014). 24966298 10.1152/jn.00052.2014 PMC4157170

[86] M. J. Arcaro, T. Mautz, V. K. Berezovskii, M. S. Livingstone, Anatomical correlates of face patches in macaque inferotemporal cortex. Proc. Natl. Acad. Sci. **117**, 32667–32678 (2020). 33277435 10.1073/pnas.2018780117 PMC7768718

[87] T. Lin , “Microsoft COCO” in Common Objects in Context in European Conference on Computer Vision, T. Fleet, D. Pajdla, T. Schiele, B. Tuytelaars, Eds. (Springer, 2014), pp. 740–755.

[88] M. J. Arcaro, M. S. Livingstone, A hierarchical, retinotopic proto-organization of the primate visual system at birth. eLife **6**, 1–24 (2017). 10.7554/eLife.26196 PMC5495573 28671063

[89] W. Vanduffel , Visual motion processing investigated using contrast agent-enhanced fMRI in awake behaving monkeys. Neuron **32**, 565–577 (2001). 11719199 10.1016/s0896-6273(01)00502-5

[90] S. Sharma, D. J. Schaeffer, K. Vinken, S. Everling, K. Nelissen, Intrinsic functional clustering of ventral premotor F5 in the macaque brain. Neuroimage **227**, 117647 (2021). 33338618 10.1016/j.neuroimage.2020.117647

